# Structural basis of methotrexate and pemetrexed action on serine hydroxymethyltransferases revealed using plant models

**DOI:** 10.1038/s41598-019-56043-4

**Published:** 2019-12-23

**Authors:** Milosz Ruszkowski, Bartosz Sekula, Agnieszka Ruszkowska, Roberto Contestabile, Isabel Nogues, Sebastiana Angelaccio, Andrzej Szczepaniak, Zbigniew Dauter

**Affiliations:** 1 Synchrotron Radiation Research Section of MCL, National Cancer Institute, Argonne, IL USA; 2 Institute of Bioorganic Chemistry, Polish Academy of Sciences, Poznan, Poland; 3 Department of Chemistry and Biochemistry, University of Notre Dame, Notre Dame, IN USA; 4 Department of Biochemical Sciences “A. Rossi Fanelli”, Sapienza University of Rome, Laboratory affiliated to Istituto Pasteur Italia-Fondazione Cenci Bolognetti, P.le Aldo Moro 5, 00185 Roma, Italy; 5 Research Institute of Terrestrial Ecosystems, Italian National Research Council, Via Salaria Km. 29, 300–00015 Monterotondo Scalo, Roma Italy; 6 Department of Hematology and Bone Marrow Transplantation, Poznan University of Medical Sciences, Poznan, Poland

**Keywords:** X-ray crystallography, Transferases

## Abstract

Serine hydroxymethyltransferases (SHMTs) reversibly transform serine into glycine in a reaction accompanied with conversion of tetrahydrofolate (THF) into 5,10-methylene-THF (5,10-meTHF). *In vivo*, 5,10-meTHF is the main carrier of one-carbon (1C) units, which are utilized for nucleotide biosynthesis and other processes crucial for every living cell, but hyperactivated in overproliferating cells (e.g. cancer tissues). SHMTs are emerging as a promising target for development of new drugs because it appears possible to inhibit growth of cancer cells by cutting off the supply of 5,10-meTHF. Methotrexate (MTX) and pemetrexed (PTX) are two examples of antifolates that have cured many patients over the years but target different enzymes from the folate cycle (mainly dihydrofolate reductase and thymidylate synthase, respectively). Here we show crystal structures of MTX and PTX bound to plant SHMT isozymes from cytosol and mitochondria—human isozymes exist in the same subcellular compartments. We verify inhibition of the studied isozymes by a thorough kinetic analysis. We propose to further exploit antifolate scaffold in development of SHMT inhibitors because it seems likely that especially polyglutamylated PTX inhibits SHMTs *in vivo*. Structure-based optimization is expected to yield novel antifolates that could potentially be used as chemotherapeutics.

## Introduction

**Figure 1:**
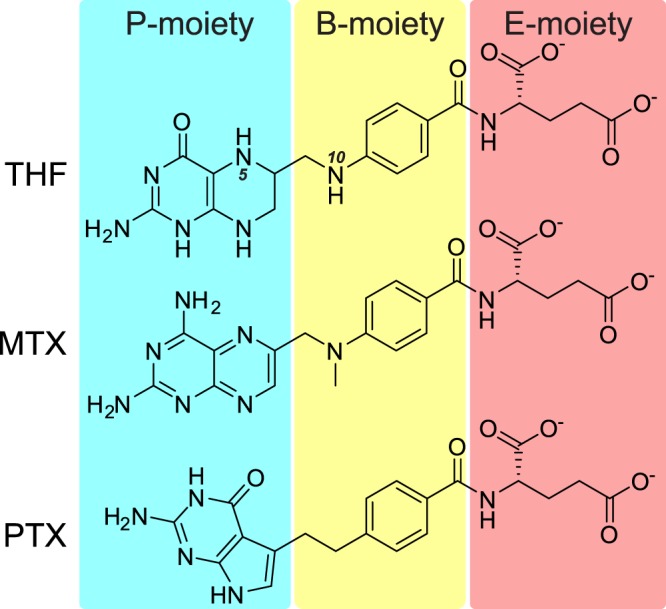
Structural formulas of tetrahydrofolate (THF), methotrexate (MTX), and pemetrexed (PTX). The compounds are divided into P-, B-, and E-moieties, as referenced throughout the text.

Serine hydroxymethyltransferase (SHMT) reversibly interconverts serine and tetrahydrofolate (THF, Fig. 1) into glycine and 5,10-methylene-THF (5,10-meTHF)^1,2^. As a part of one-carbon (1C) metabolism, SHMTs fuel the folate cycle by providing 5,10-meTHF^3^. The folate cycle is responsible for biotransformation of folate species: THF, 5,10-meTHF, 5-methyl-THF, and 10-formyl-THF, with each of them playing distinct and crucial cellular roles. Intracellular membranes appear impermeable to 1C-loaded THF species—hence the need to generate 5,10-meTHF separately in subcellular compartments^4^. In mammals, SHMT isoforms operate in cytosol (e.g. human cytosolic SHMT (hcSHMT or SHMT1); UniProt ID: P34896) and mitochondria (human mitochondrial SHMT (hmSHMT or SHMT2); UniProt ID: P34897). Moreover, direction of the SHMT-catalyzed reaction is controlled independently in organelles *via* supply and demand of 1C units^5^. Therefore, depending on the metabolic status on cellular and subcellular levels, serine can be either catabolized or anabolized by SHMTs with simultaneous production or degradation of 5,10-meTHF^5^. Interestingly, plant genomes encode additional isozymes that localize in chloroplasts and nuclei^6,7^. It has been shown that in photorespiration the flux of serine-to-glycine interconversion is shifted towards serine synthesis that is thermodynamically less favored^8,9^. Increased activity of the glycine cleavage system (GCS) in the mitochondrial matrix results in high 5,10-meTHF/THF ratio^10–12^. This way, the SHMT-catalyzed reaction is one of the three pathways used by plants for serine biosynthesis^13^.

In general, 1C-related enzymes have been actively studied for many years, mainly because 1C units are utilized in crucial biosynthetic pathways, such as purine and thymidine biosynthesis, regeneration of S-adenosyl methionine, etc. These processes are vital to all living cells but in overproliferating cells (e.g. cancer tissues) operate at enhanced rates^14,15^. Therefore, by inhibiting SHMTs, it appears possible to limit the supply of 1C units, which are in highest demand by cancer cells. Such approach would impact not only the aforementioned biosynthetic pathways, but through them, would also influence epigenetic maintenance, amino acid homeostasis, and redox defense.

Methotrexate (MTX) (Fig. 1), a methylated derivative of aminopterin, was the first selective chemotherapeutic targeting 1C metabolism. Since its introduction in 1950^16^, the use of MTX has gradually increased and now it is used to cure millions of cancer patients annually. MTX treatment, as a single agent or as a part of multidrug chemotherapy, is effective against leukemia, non-Hodgkin lymphomas—particularly with central nervous system involvement—and a number of other cancer types^17^. As a chemotherapeutic MTX is used in high doses over a short period of time and its efficacy depends on the duration of infusion and on intracellular drug concentration. Due to its very poor ability to cross the blood-brain barrier, MTX is administered at remarkably high doses to obtain therapeutic concentrations in the tumor tissue and in lymphoma-surrounding neural tissue^18^. Of course, such toxic levels of the drug also impact non-cancer cells. Therefore, MTX treatment is followed by folate supplementation to the patient until blood concentration of MTX becomes sufficiently low. Additionally, to limit systemic exposure, intra-arterial, intrathecal or intraventricular delivery is used to reach higher concentration of the drug in the central nervous system^19^. In low doses, MTX is generally well tolerated, and prolonged low-dose therapy is given to patients suffering from autoimmune diseases, such as lupus, psoriasis, sarcoidosis, and rheumatoid arthritis^20^.

Pemetrexed (PTX) is a relatively new drug, introduced in early 2000s^21,22^. From the chemical standpoint, PTX (unlike MTX) lacks the pteridine ring (Fig. 1). Non-small cell lung cancer and pleural mesothelioma are the only approved indications for PTX usage; however, trials are currently under way for testing it against other cancers (e.g. esophageal squamous cell carcinoma)^23^. Similarly to the MTX therapy, patients taking PTX are usually also supported with folate.

MTX and PTX inhibit folate-related enzymes. Specifically, MTX is most potent against dihydrofolate reductase (DHFR), whereas PTX targets mostly thymidylate synthase (TS) but also 5-aminoimidazole-4-carboxamide ribonucleotide transformylase (AICART), and glycinamide ribonucleotide formyltransferase (GARFT)^24,25^. However, there have been implications that MTX and PTX inhibit SHMTs as well^26^. This is not surprising, considering that MTX and PTX are chemically similar to THF, although they are not able to accept 1C units. However, because of chemical differences, it was rather obvious that binding of MTX, PTX, and THF by SHMTs should be different. Since SHMT enzymes are promising targets for drug design the need for such structures is quite apparent, as emphasized by an attempt to model the interactions by *in silico* docking^26^. Importantly, computational drug design methods, which minimize the cost of development and increase the chance of finding a good candidate, are most successful if a crystal structure is known^27^. After completion of experiments for this article, structures of hmSHMT in complexes with PTX and lometrexol showed first structural insights into SHMT interactions with antifolates^28^. However, it has been impossible to obtain structures of MTX bound to human SHMT isoforms or any structure showing a cytosolic isoform complexed with antifolate. Our recent work on plant SHMTs^7^ inspired us to use plant models, as we found that two *Arabidopsis thaliana* isoforms produce crystals diffracting to much higher resolution than the human isozymes. The two *A. thaliana* enzymes: mitochondrial *At*SHMT2 (UniProt ID: Q94C74) and cytosolic *At*SHMT4 (UniProt ID: O23254) are counterparts of hmSHMT and hcSHMT, respectively (of corresponding subcellular localizations). Amino acid sequence identities between corresponding plant and human isoforms are 60%, whereas hmSHMT and hcSHMT share 66% identity, and *At*SHMT2 and *At*SHMT4 share 56% identity (excluding mitochondrial target peptides).

In this work, we have performed functional *in vitro* studies using purified isozymes to investigate whether the behavior of cytosolic *At*SHMT4 resembles that of hcSHMT, and if mitochondrial *At*SHMT2 is similar to hmSHMT. Next, we have obtained high resolution crystal structures of *At*SHMT2 complexed with MTX and PTX, as well as of *At*SHMT4-MTX complex. Detailed analysis of the interacting residues and the environment in and near the binding cavities allowed us to indicate key structural elements responsible for antifolate binding. We have found features that are either similar or vary between MTX and PTX complexes. Comparisons of amino acid sequences and structures of the binding sites show that our results very likely apply to the human SHMTs as well. In our view, these observations should stimulate design of SHMT-targeted antifolates. Quoting Sir James Whyte Black: “the most fruitful basis for the discovery of new drug is to start with an old drug”^29,30^. We hope to utilize MTX and PTX as starting points for the development of novel chemotherapeutics targeted at SHMTs. Such an attempt will benefit from the fact that starting molecules are already approved drugs and have desired or acceptable ADME-Tox properties (absorption, distribution, metabolism, excretion, and toxicity). Therefore, it is expected that agents based on MTX and/or PTX but targeted at SHMT will become valuable.

## Results

### Dependence of *At*SHMT2 and *At*SHMT4 activity on pH is similar to that of their human counterparts of corresponding subcellular localizations

**Figure 2:**
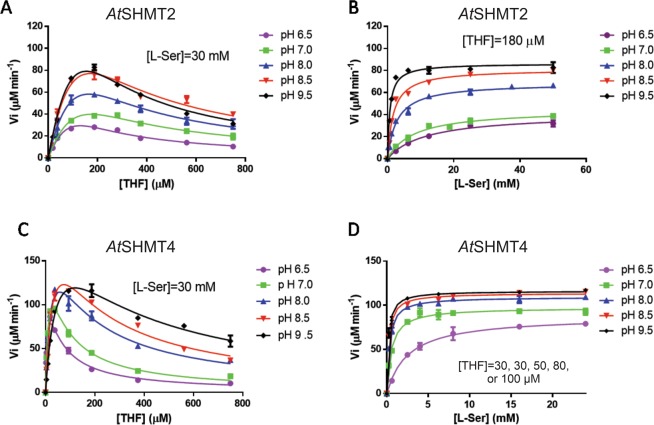
Dependence of *At*SHMT2 and *At*SHMT4 activity on pH. The initial velocity of the SHMT forward reaction was measured at different pH values with *At*SHMT2 and *At*SHMT4 (0.2 μM) using a fixed concentration of one substrate while varying the concentration of the other substrate. The fixed L-serine concentration was saturating. THF fixed concentration was giving the maximum activity when varying L-serine. All experimental points are the average ± standard deviation of three independent measurements. Panel (A) *At*SHMT2, 30 mM L-serine; Panel (B) *At*SHMT2, 180 µM THF; Panel (C) *At*SHMT4, 30 mM L-serine; Panel (D) *At*SHMT4, 30 µM THF at pH 6.5 and 7.0, 50 µM THF at pH 8.0, 80 µM THF at pH 8.5 and 100 µM THF at pH 9.5. Reactions were carried out at 30 °C.

Measurements of the initial velocity of the SHMT forward reaction (serine + THF → glycine + 5,10-meTHF) were carried out with *At*SHMT2 and *At*SHMT4 at different pH values, keeping one substrate at fixed concentration while varying the concentration of the other substrate (Fig. 2). As with all other characterized SHMTs isolated from different sources^31^, a clear THF substrate inhibition is visible (Table 1). The behavior of *A. thaliana* SHMTs is similar to that of human isoforms. The THF substrate inhibition constant (*K*_i_) with human and plant mitochondrial SHMTs (hmSHMT and *At*SHMT2, respectively) stays more or less constant as pH is varied, whereas this parameter is greatly increased as pH increases with human and plant cytosolic isoforms (hcSHMT and *At*SHMT4). Moreover, with both *At*SHMT2 and *At*SHMT4, *K*_M_ for L-serine decreases as pH is increased (Table 1). This characteristic is in common with human SHMT isoforms^31^. Dependence of *K*_M_ for THF is also similar between human and *A. thaliana* SHMT isoforms: this parameter stays constant with the mitochondrial isoforms and increases as pH is increased with the cytosolic isoforms. With both *A. thaliana* isoforms, *k*_cat_ increases as pH is increased. In this respect, *At*SHMT4 is different from the human cytosolic isoform that shows an opposite behavior^32^. This discrepancy in behavior might be due to the different experimental conditions used to characterize the enzymes, which include buffering compounds and salts that impact ionic strength. *At*SHMT2 is also somewhat different from hmSHMT, for which *k*_cat_ stays constant between pH 6.9 and 7.8 pH and increases when pH is increased to 8.4^31^. Unfortunately, based on current data we cannot provide rational explanations of such differences between human and *Arabidopsis* SHMTs.

**Table 1:** Steady-state kinetic parameters of the SHMT forward reaction with *At*SHMT2 and *At*SHMT4.

	pH	*K*_M_		*k*_cat_ (s^−1^)	*k*_cat_/*K*_M_		*K*_i_THF (μM)
		L-Ser (mM)	THF (μM)		L-Ser	THF	
*At*SHMT2	6.5	11.65 ± 2.03	83.6 ± 8.1	5.3 ± 0.4	0.45	0.06	189.4 ± 12.8
	7	8.05 ± 1.36	153.6 ± 10.9	8.1 ± 0.7	1.01	0.05	191.3 ± 9.5
	8	2.86 ± 0.30	143.9 ± 12.6	13.0 ± 0.7	4.53	0.09	186.4 ± 11.7
	8.5	1.78 ± 0.22	196.1 ± 14.6	20.6 ± 1.3	11.57	0.11	151.8 ± 8.7
	9.5	0.71 ± 0.09	192.4 ± 10.4	21.8 ± 2.3	30.73	0.11	127.4 ± 5.5
*At*SHMT4	6.5	2.56 ± 0.26	7.87 ± 1.62	11.9 ± 1.3	4.63	1.51	48.13 ± 9.69
	7	0.54 ± 0.05	14.97 ± 1.87	16.2 ± 1.1	29.97	1.08	57.49 ± 6.56
	8	0.30 ± 0.02	23.83 ± 3.99	17.4 ± 1.7	57.89	0.73	141.0 ± 23.23
	8.5	0.24 ± 0.02	33.75 ± 5.63	19.6 ± 1.9	81.53	0.58	163.3 ± 25.96
	9.5	0.20 ± 0.02	49.24 ± 6.62	18.2 ± 1.4	90.75	0.37	288.1 ± 39.18

### Methotrexate and pemetrexed inhibit mitochondrial *At*SHMT2 and cytosolic *At*SHMT4

Because of the specific activity assay used to characterize the inhibition by PTX and MTX, we needed a full characterization of the equilibrium binding between SHMT, glycine and 5-formyl-THF. This was carried out by measuring the absorbance change at around 500 nm observed when SHMT binds both glycine and the folate ligand forming a quinonoid intermediate. From a series of experiments, in which 5-formyl-THF was varied as glycine was kept fixed at different concentrations (Fig. S1), *K*_d_ and α*K*_d_ were determined, which are respectively the dissociation constant of one ligand in the absence of the other and the dissociation constant of one ligand in the presence of a saturating concentration of the other ligand. We noticed that the formation of the quinonoid with *At*SHMTs was much slower than with the human SHMT isoforms (data not shown).

**Figure 3:**
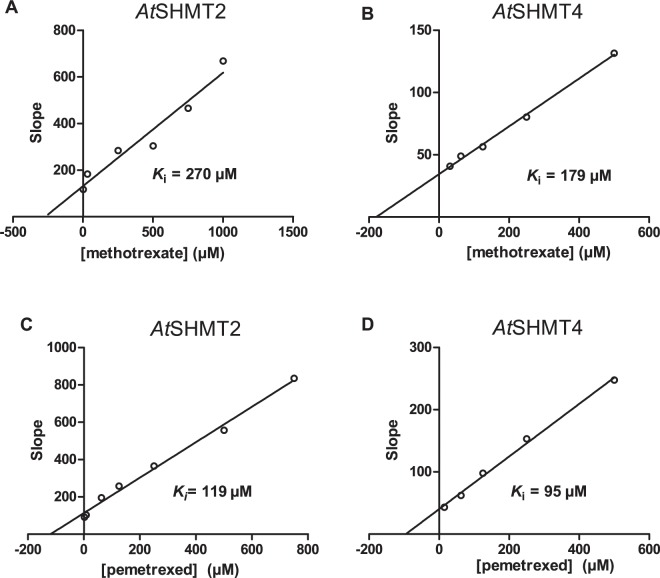
*At*SHMT2 and *At*SHMT4 inhibition by MTX and PTX. Panel (A) shows data for *At*SHMT2 inhibition by MTX; (**B**) *At*SHMT4 by MTX; C, *At*SHMT2 by PTX; D *At*SHMT4 by PTX. Secondary plots of slopes as functions of antifolate concentrations were obtained from fitting of inhibition data (Fig. S2). Intercept on the X-axis gives an estimate of the inhibition constant (*K*_i_) related to antifolate binding to the enzyme-glycine complex. Since it is known that the mitochondrial matrix has an alkaline pH (pH 8^68^) as compared to the cytosol (pH 7.3^68^), inhibition measurements were performed in 20 mM KPi buffer at pH 8.0 for *At*SHMT2 and pH 7.3 for *At*SHMT4 at 30 °C. Measurements were carried out varying 5-formyl-THF concentration, while keeping glycine at 10 mM and antifolates at different fixed concentrations. Units of slopes are μM.

MTX and PTX inhibition was analyzed in competitive binding assays, in which 5-formyl-THF and the antifolate compete for binding to SHMT. In this assay, the absorbance at 500 nm of the quinonoid is measured at variable 5-formyl-THF concentrations and glycine is kept at a fixed, saturating concentration (10 mM) while antifolate is fixed at different concentrations (Figs. 3, S2). In this system, the obtained *K*_i_ values refer to binding of the antifolates to the enzyme-glycine complex. The *K*_i_ values obtained for human and plant SHMTs are compared in Table 2.

**Table 2:** Methotrexate and pemetrexed Inhibition constant values obtained with human cytosolic (hcSHMT) and plant SHMT isoforms.

SHMT isoform	*K*_i_MTX (µM)	*K*_i_PTX (µM)
hcSHMT	^a^201.0 ± 19	^b^19.1 ± 3.1
*At*SHMT4	179.3 ± 9.3	95.1 ± 9.3
*At*SHMT2	270.2 ± 62.5	119.4 ± 13.2

^a^Apparent inhibition constant from Paiardini *et al*.^45^.

^b^Inhibition constant from Daidone *et al*.^26^.

### Methotrexate binding mode

From structures obtained by soaking *At*SHMT2 and *At*SHMT4 crystals with MTX we learn that the antifolate can bind in three distinct ways. At this point it is also worth to mention that each pose of antifolate (MTX and PTX) was verified by calculating Polder maps^33^ and by analysis of the map shapes and correlation coefficients. For clarity, in this work we divide THF, MTX, and PTX molecules into three moieties: (i) pteridine or its mimic (P-moiety, Fig. 1); (ii) benzoyl (B-moiety); and (iii) glutamate (E-moiety).

**Figure 4:**
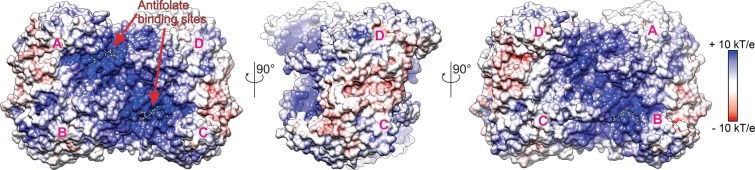
Electrostatic potential and antifolate binding site. *At*SHMT2-MTX complex (tetramer) is shown with marked subunits (**A–D**).

**Figure 5:**
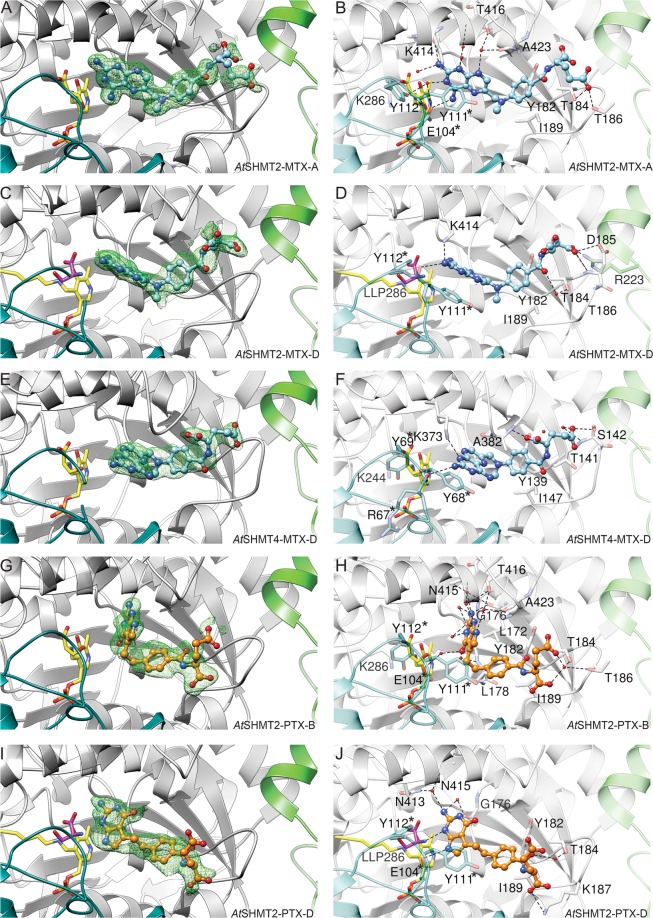
MTX and PTX binding by SHMTs. Green mesh (in **A,C,E,G,I**) represents Polder electron density maps contoured at 4.5 σ calculated for 5 Å volume around the antifolates. Panels (B,D,F,H,J) show networks of interactions between SHMTs and antifolates. Internal (LLP) or external aldimines are yellow, free serine is in magenta, residues from another protein subunit than indicated in caption, involved in interactions with MXT or PTX, are marked by asterisks.

Both *At*SHMT2 and *At*SHMT4 are tetramers based on size-exclusion chromatography. The subunits are marked A-D (counterclockwise in Fig. 4). Interfaces between subunits A/B and C/D are much more extensive than e.g. between A and D. In the crystal structure of *At*SHMT2-MTX complex, we noted two binding modes of MTX, one corresponding to external PLP-Ser aldimine state (EA-type; chains A, B, C) and the other to PLP internal aldimine + free serine state (IA-type; chain D). At this moment, we can only assume that different reaction states result from different neighborhood of subunits in the crystal lattice and therefore different access to solvent channels—although cooperativity between subunits cannot be excluded. The EA-type of binding (Fig. 5A,B) will be described based on chain A due to the higher quality of electron density maps and the lower B-factors. In this case, direct hydrogen bonds dock exocyclic N4 of MTX to Oε of Glu104* (an asterisk indicates a residue from another, tightly bound subunit), and N2 of MTX to backbone O of Lys414. A network of water-mediated hydrogen bonds link the antifolate to Thr416, Ala423, and Glu104*. Altogether, five out of six heteroatoms of the P-moiety (pteridine-2,4-diamine) participate in hydrogen bonding. Tyr111* π-stacks with the pteridine ring. B-moiety is sandwiched by the side chains of Ile189 and Tyr182 from one side and Ala423 from the other side. Finally, E-moiety is mostly disordered but appears to point towards the residues 184–186, with Oε of the E-moiety interacting with Oγ of Thr186.

The IA-type binding mode (Fig. 5C,D; chain D) is characterized by fewer H-bonds. In the P-moiety, exocyclic N2 H-bonds to carbonyl O of Tyr111* and to Nζ of Lys414. The pteridine ring is again stacked with the side chain of Tyr111*, however, it is rotated by 66° with respect to the ring in the EA-type binding. Surroundings of the B-moiety are similar to those observed in the EA-type, but the benzoyl ring is tilted by 23°. The benzoyl O atom interacts with Oγ of Thr184 *via* a water molecule. The two Oε atoms of the E-moiety are also involved in binding; Oε1 to the backbone amide of Asp185 and to (apparently protonated) Oδ of Asp185, whereas Oε2 interacts with Nη of Arg223 from the A-chain.

In *At*SHMT4, (Fig. 5E,F, chain D) N1 of pteridine-2,4-diamine (with *sp*^3^ hybridization) interacts with Nζ of Lys373, whereas exocyclic N2 binds to carbonyl O of Arg67. The pteridine ring is stacked with the side chain of Tyr68*, equivalent to Tyr111* in *At*SHMT2. The B-moiety is shielded by Ile147, Tyr139, and Ala382, corresponding to Ile189, Tyr182, and Ala423 of *At*SHMT2, respectively. The E-moiety interacts with the backbone amide of Ala382 and Oγ of Ser142. Despite most of the interacting residues being conserved in *At*SHMT2 and *At*SHMT4, positioning of the P-moiety of MTX is different. In *At*SHMT4, all four subunits within the crystal structure are in the external aldimine PLP-Ser form. Therefore, it is striking to see that the P-moiety in *At*SHMT4 structure is posed in the same plane but rotated 180° with respect to the IA-type in *At*SHMT2. Consistently, the stacking Tyr68* has a conformation similar to that of the corresponding Tyr111* of *At*SHMT2 in the IA-type, and not to the EA-type as expected. This conformation of Tyr68* is even more striking when we consider that conformation of Tyr69* (*At*SHMT4) is very different from that of Tyr112* in *At*SHMT2 (IA-type).

### Pemetrexed binding mode

Soaking crystals of *At*SHMT2 with PTX and serine revealed two distinct forms, resembling situation observed for *At*SHMT2-MTX complex. External PLP-Ser aldimine state (EA-type) is seen in chains B and C (PTX was not placed in chain A as suggested by poor Polder map coefficients^33^). Internal aldimine + free serine state (IA-type) is present in chain D.

In the EA-type of binding (Chain B, Fig. 5G,H), a direct H-bond binds exocyclic N2 of PTX to backbone O of Leu172. Water-mediated interactions involve each of the five heteroatoms in the P-moiety. Specifically, N1 is bonded to backbone N and Oγ of Thr416; N2 to Nδ of Asn415 and backbone O of Gly176; N3 to backbone O of Gly176; O4 to backbone N of Leu178; N7 to Oγ of Thr416 and carbonyl O of Ala423. Importantly, there is no π-stacking interaction involving PTX. Hydrophobic environment is ensured mostly by side chains of Leu172, Leu178, and Ile189 adjacent to PTX B-moiety. The linker between P- and B-moieties is shielded by the ring of Tyr111*. The E-moiety is partially disordered and located next to the loop fragment _184_TDTK_187_, interacting with Oγ of Thr184 and Oγ of Thr186 via a water molecule. The carboxyl group (at Cα) forms a salt bridge with Nζ of Lys187.

In the IA-pose (Chain D, Fig. 5I,J), PTX forms fewer H-bonds. N1 directly H-bonds N of the free serine; N2 binds via water molecules to Nδ of Asn413, Oδ of Asn415, carbonyl O of Gly176 and Leu172; O4 is linked to Oγ of Thr416 via H_2_O; N7 directly docks to Oε of Glu104*. Like in the EA-pose, there is no π-stacking interaction of the ring moieties. The salt bridge to Lys187 is also conserved.

## Discussion

**Figure 6:**
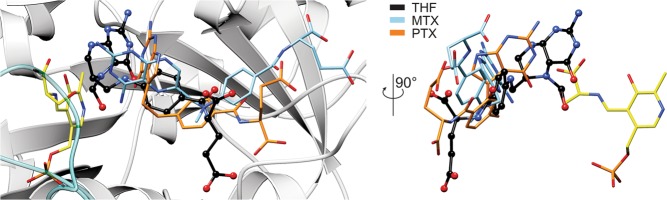
Comparison of MTX, PTX, and THF binding modes. Positioning of THF (black, ball-and-stick) was acquired by superposing THF complex with murine SHMT (PDB ID: 1eji; chain A^69^). EA-poses are shown for *At*SHMT2-MTX complex (chain A), and *At*SHMT2-PTX complex (chain B).

Although having a similar scaffold, THF, MTX, and PTX are remarkably different from the chemical standpoint. Their P-moieties show highest variability (Fig. 1). MTX and PTX have dual-ring aromatic P-moieties, in contrast to THF. This is the most profound difference, making MTX and PTX unable to accept 1C units. The P-moieties of MTX and PTX vary significantly, too. PTX has a six-membered ring fused with a five-membered ring. As a consequence, it lacks what would be the N5 atom of MTX. It is therefore interesting to see that in the SHMT-MTX complexes, N5 never interacts with the protein. Removing an unnecessary H-bond donor seems like a step towards higher specificity and agrees with our kinetic data. Another feature that likely explains higher inhibitory efficiency of PTX against SHMTs is that it is able to bind deeper inside the cavity (Fig. 6). When we look at the P-moieties as if they were nucleobases, we see that MTX and PTX molecules (Fig. 1) have different “Watson-Crick” faces resulting in their different ability to be H-bond donors/acceptors. In this context, PTX is a closer mimic of THF. This explains why PTX can penetrate deeper into the cavity (Fig. 6) and be a more potent inhibitor of SHMTs (Table 2).

**Figure 7:**
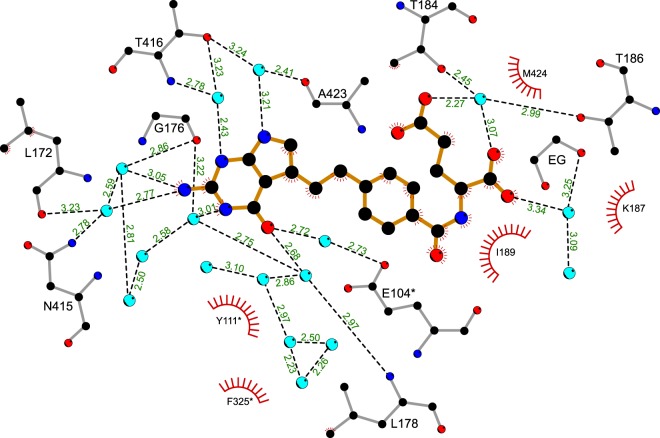
Detailed binding mode of PTX to *At*SHMT2. PTX (orange bonds) interacts with the protein *via* an extensive network of hydrogen bonds (dashed, black lines; distances are given in Å), mostly mediated by water molecules (light blue circles). Hydrophobic interactions are indicated by “eyelashes”. Chain B of *At*SHMT2-PTX complex is shown; EG, ethylene glycol molecule.

The antifolate binding presented in this work shows that water molecules play a vital role in creating the extensive network of hydrogen bonds (Figs. 5 and 7). We propose to utilize positions of those water molecules (with respect to antifolates) as placeholders for specific functional groups which could be either H-bond acceptors or donors. This approach could likely enhance specificity and inhibitory properties of antifolates against SHMTs. It is also very important to note that resolving positions of water molecules inside the antifolate-binding cavity was possible due to high resolution diffraction data obtained particularly for *At*SHMT2 structures. Lower resolution data from hmSHMT crystals^28^ (2.3 Å, Protein Data Bank, PDB ID: 6qvl) allowed to trace only a very limited number of water molecules.

**Figure 8:**
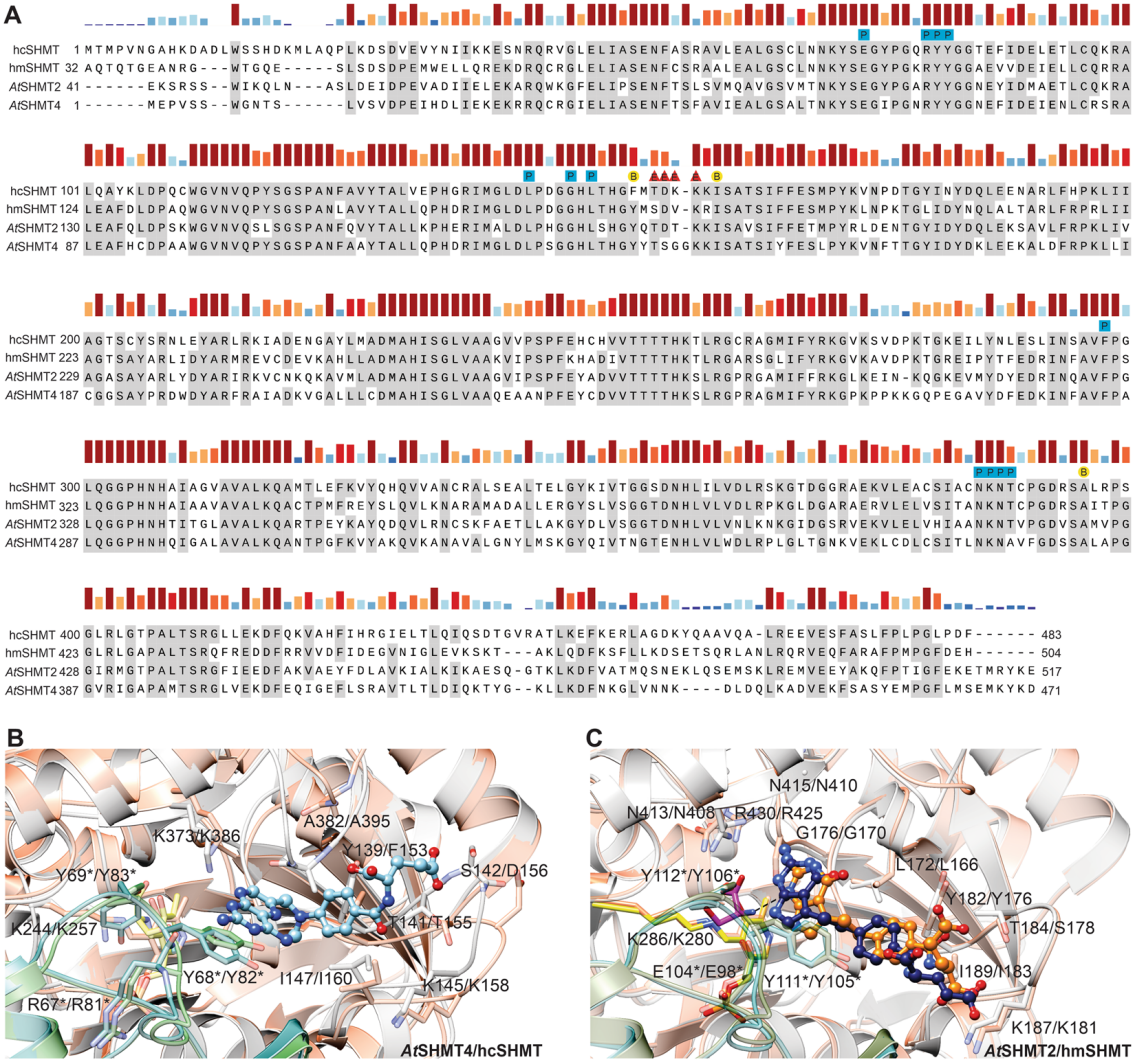
Comparison of hcSHMT, hmSHMT, *At*SHMT2, and *At*SHMT4. In Panel (A), bars above the sequences indicate residue conservation. Residues interacting with P-, B-, and E-moieties are marked with blue squares, yellow circles, and red triangles, respectively, and labeled with a corresponding letter. Panels (B,C) show pairwise structural comparisons of cytosolic *At*SHMT4 (gray and teal subunits; blue MTX) with hcSHMT (coral, green), and of mitochondrial *At*SHMT2 (gray, teal; orange PTX) with hmSHMT (coral, green; navy PTX). Residues and their numbering are given for plant/human isoforms. Antifolates are shown in ball-and-stick representation.

Considering the three moieties (P, B, and E) as pharmacophores, it is worth looking at the sequence and structure conservation between human and plant SHMTs with respect to regions of pharmacophore binding (Fig. 8). The sequence alignment shows a nearly complete conservation of residues that bind P and B moieties. Variability is present in the E-moiety binding region, however, the residue that ensures positive charge of the loop (Lys145 in *At*SHMT4 and Lys187 in *At*SHMT2) is universally conserved. The sequence-based conclusions are supported by structural superpositions (Fig. 8B,C). More precisely, for hcSHMT structure^34^ without a folate (PDB ID: 1bj4; RMSD = 0.75 Å with *At*SHMT4-MTX) positions of most ligand-interacting residues are conserved with exceptions of loops containing Ala395 and Lys158 of hcSHMT that are in “open conformations” (Fig. 8B). It is likely, however, that upon folate binding the loops close and their conformations resemble those of *At*SHMT4-MTX complex. Comparison of the mitochondrial isoforms, hmSHMT (PTX complex, PDB ID: 6qvl) with *At*SHMT2-PTX (chain D, RMSD = 0.56 Å) shows even more similarities. Strikingly, for most residues involved in interaction with antifolates not only positions but also side chain conformations are the same (Fig. 8C). A deeper insight into PTX binding mode presented in *At*SHMT2-PTX complex revealed that PTX pose in chain D is very similar to that presented in hmSHMT-PTX complex (PDB ID: 6qvl, Fig. 8C). P- and B-moieties of PTX are bound alike and small (~0.5 Å) discrepancies can likely be due to free serine that is bound to *At*SHMT2 in addition to PTX. In fact, this free serine is involved in interaction between the protein and PTX. This means that the two complexes likely represent different PTX binding scenarios, (i) a pre-catalytic state at high serine concentration (*At*SHMT2-PTX, chain D) or (ii) in serine-deficient state (hmSHMT-PTX complex).

After their uptake inside the cell by the reduced folate carrier^35,36^, MTX and PTX undergo polyglutamylation by ATP-dependent folylpoly-γ-glutamate synthetase (FPGS), analogously to the folate species^37–40^. Polyglutamylation renders folates (or antifolates) polyanions that are not recognized by cellular efflux systems, and therefore accumulate in cellular compartments^41^. More importantly, polyglutamylated folates and antifolates present increased binding affinity to the folate-dependent enzymes. It has been shown that pentaglutamylated PTX is roughly 100-fold more potent than its monoglutamyl form; for instance, *K*_i_ against TS decreases from 109 nM to 1.3 nM, whereas *K*_i_ against GARFT decreases from 9.3 μM to 65 nM^42^. Notably, the *K*_i_ value of PTX against hcSHMT of 19.1 μM is very close to *K*_i_ against GARFT^26^. Intracellular concentration of PTX (or its polyglutamylated forms) reach 10–30 μM^43^. This suggests that polyglutamylated PTX may already effectively inhibit hcSHMT *in vivo*.

The vital role of polyglutamylation is consistent with the fact that loss of FPGS activity in patients, usually due to recurrent exposure, causes resistance to antifolates such as MTX and PTX^38,44^. Higher inhibitory activity of polyglutamylated antifolates against TS and GARFT allows us to hypothesize that the *in vivo* activity against SHMTs is also significantly higher. This hypothesis is supported by the fact that in the case of *At*SHMT3, *K*_M_ for THF decreases ~340-fold (218 μM for THF, and 0.64 μM for tetraglutamylated THF)^6^. Although comparing *K*_i_ and *K*_M_ values can be misleading, higher affinity for polyglutamylated antifolates appears clear. It is also consistent with location of the positively-charged surface area around the entrance to the antifolate binding cavity that is able to attract negatively-charged polyglutamyl tail of THF or antifolates (Fig. 4). Unfortunately, polyglutamated forms of MTX or PTX are currently not commercially available, thus we cannot verify whether they indeed inhibit SHMTs much stronger than the non-glutamylated forms. So far, research on anticancer drugs has yielded candidate scaffolds for future drugs^45^. Pyrazolopyran-based inhibitors are effective against hcSHMT^46^. However, search for new antitumor agents by no means has been the only attempt to target SHMTs. For instance, an extensive drug design campaign is aimed at SHMTs from a malarial parasite *Plasmodium falciparum* SHMTs^47–50^. Inhibitors based also on pyrazolopyran showed a remarkable potency against *Pf*SHMT^48^. One of the inhibitors was able to reduce parasitemia *in vivo* in a mouse model by 73%^49^. Herbicidal and pharmaceutical use of pyrazolopyrans is a subject of a patent (WO2013182472A1). So far, low metabolic stability limits the use of many potent SHMT inhibitors^48^. In this context, exploiting commonly used drugs (with some inhibitory activity against SHMTs) as starting scaffolds appears to be a very auspicious concept.

## Conclusions and Future Outlook

In light of recent discoveries, design of SHMT inhibitors seems to be promising in finding novel chemotherapeutics. However, we are challenged by (i) high dynamics of SHMTs, related to the reaction state, (ii) apparently multiple binding modes of antifolates, and (iii) the need for candidate drugs to be polyglutamylated by FPGS. Therefore, we are very optimistic to note that already available antifolate chemotherapeutics, such as PTX, are potent against SHMT enzymes. In our opinion, further optimization of candidate drugs, for instance by adding functional groups positioned as water molecules in our complexes has a very high chance of success. With that in mind, we believe that our results have just brought us a step closer towards a rationalized targeting of SHMTs.

## Methods

### Cloning, overexpression, and purification of *At*SHMT2 and *At*SHMT4

*At*SHMT2 and *At*SHMT4 were produced using a protocol similar to that for the production of *Medicago truncatula* SHMT3^7^. Briefly, RNA isolated from *A. thaliana* leaves using the RNeasy Plant Mini Kit (Qiagen) was reverse-transcribed into the complementary DNA (cDNA) with SuperScript II reverse transcriptase (Life Technologies). The mitochondrial signal peptide of *At*SHMT2 was recognized using the TargetP 1.1 server^51,52^; the construct was designed to yield the protein N-truncated at E41. The open reading frames coding for *At*SHMT2 41–517 fragment and full-length *At*SHMT4 were amplified by polymerase chain reaction. The following primers were used:

AtSHMT2-Nt41-F, TACTTCCAATCCAATGCCGAGAAATCTCGTTCTAGTTGGATTAAGCAATT; *At*SHMT2-R, TTATCCACTTCCAATGTTACTACTCTTTGTATCTCATCGTCTCTTTCT; *At*SHMT4-F, TACTTCCAATCCAATGCCATGGAACCAGTCTCTTCATGGGGTAA; *At*SHMT4-R, TTATCCACTTCCAATGTTACTAATCCTTGTACTTCATCTCAGACATGA. Expression plasmids, based on pMCSG68 vector (Midwest Center for Structural Genomics, USA), were created by the ligase-independent cloning method^53^. The proteins were expressed in BL21 Gold *E. coli* cells (Agilent Technologies) in LB media with 150 μg/mL ampicillin. When the cultures reached A_600_ of 1.0, isopropyl-D-thiogalactopyranoside was added at a final concentration of 0.5 mM to chilled cultures (18 °C); overexpression was continued for 18 h. The cell pellet from the 2 L culture was centrifuged at 3,500 x g for 30 min at 4 °C, resuspended in 35 mL of binding buffer [50 mM Hepes-NaOH pH 7.5; 500 mM NaCl; 20 mM imidazole; 1 mM tris(2-carboxyethyl)phosphine (TCEP)], and stored at −80 °C.

The cells were disrupted by sonication and the lysates were cleared by centrifugation at 25,000 × g for 30 min at 4 °C. The supernatants were run through columns packed with 3 mL of HisTrap HP resin (GE Healthcare) connected to vacuum pump-VacMan setup (Promega). The resin-bound proteins were washed six times with 40 mL of the binding buffer. Afterwards, the proteins were eluted with 20 mL of elution buffer (50 mM Hepes-NaOH pH 7.5; 500 mM NaCl; 400 mM imidazole; 1 mM TCEP). Overnight dialysis (at 4 °C) lowered the imidazole concentration to 20 mM. Simultaneously, the His_6_-tags were cleaved with TEV protease (at the final concentration 0.1 mg/mL). A second run on the HisTrap columns was used to capture the cleaved His_6_-tags and the His_6_-tagged TEV protease. The samples were concentrated to 2.5 mL and applied on a HiLoad Superdex 200 16/60 column (GE Healthcare), equilibrated with a buffer composed of 25 mM Hepes-NaOH pH 7.5, 100 mM KCl, 50 mM NaCl, and 1 mM TCEP. The proteins were concentrated using centrifugal concentrators (Millipore): *At*SHMT2 to 15 mg/mL (based on A_280_ extinction coefficient, ε = 49,300 M^−1^ × cm^−1^); *At*SHMT4 to 19 mg/mL (ε = 54,800 M^−1^ × cm^−1^).

### Kinetic tests and inhibition studies

The SHMT activity was measured with 0.2 μM enzyme samples with L-serine and THF as substrates, using a coupled assay as previously described^54^ at 30 °C. THF (and 5-formyl-THF; see below) was kindly provided by Merck & Cie (Schaffhausen, Switzerland). Activities were measured at different pH values (6.0, 7.0, 8.5 and 9.5). For pH 6.0, 7.0 and 8.5, the assays were performed in 20 mM phosphate buffer. Measurements at pH 9.5 were performed in a mixture of 50 mM MES, 50 mM HEPES, and 50 mM CHES and the final pH was adjusted with KOH.

Inhibition assays were performed using 5 μM enzyme solutions. All assays were carried out at 30 °C in 20 mM KPi buffer at either pH 8.0, in the case of *At*SHMT2, or pH 7.3 in the case of *At*SHMT4. Methotrexate was dissolved in pure DMSO. In all inhibition assays, the final concentration of DMSO was 5% (v/v), which did not affect enzyme activity^45^. Pemetrexed was dissolved in buffer.

Inhibition assays that used glycine and 5-formyl-THF were based on the spectrophotometric measurement of the quinonoid intermediate^55^. The intermediate develops when both ligands bind to SHMT, forming an enzyme-glycine-folate ternary complex. First, dissociation constants of glycine and 5-formyl-THF were determined by varying one ligand while keeping the other at a fixed and saturating concentration. Data were analyzed to obtain estimates of the dissociation constant (*K*_*d*_) and *αK*_*d*_ (in the presence of a saturating concentration of the other ligand) for both ligands, as reported in^31^.

Kinetic parameters (Table 1) were determined using Eq. 1 when L-serine was the fixed substrate and Eq. 2 when THF was the fixed substrate. In the case of data obtained with *At*SHMT2 and L-serine as fixed substrate, V_max_ was previously estimated with a normal Michaelis-Menten equation leaving out data obtained with THF concentrations higher than 100 μM; estimated V_max_ values were then used as fixed parameters when fitting all data to Eq. 1.

1 $${V}_{i}={V}_{max}\frac{[THF]}{[THF]+{K}_{m}THF(1+\frac{[THF]}{{K}_{i}})}$$

2 $${V}_{i}={V}_{max}\frac{[Ser]}{[Ser]+{K}_{m}Ser}$$

Binding of MTX and PTX to SHMT was subsequently analyzed by measuring the inhibitory effect of these compounds on the formation of the quinonoid intermediate. In the assays, 5-formyl-THF was added as the last component and, after rapid manual mixing, the absorbance change was measured at 500 nm (*At*SHMT4) or 505 nm (*At*SHMT2). The obtained data were used to produce double reciprocal plots and fitted to linear equations. Slopes of the straight lines were plotted versus MTX or PTX concentration in secondary plots and fitted to linear equations in order to find the related inhibition constants from the intercepts to the X-axes. Fitting of data was performed with the software PRISM (GraphPad, La Jolla, CA, USA). All experiments were performed in triplicate using a Hewlett-Packard 8453 diode-array spectrophotometer (Agilent Technologies, Santa Clara, CA).

### Crystallization and diffraction data collection

The *At*SHMT2 crystals were grown by vapor diffusion method in hanging drops containing 3 µL of the protein solution and 2 µL of reservoir solution. The reservoir solution was composed of 90% Hampton Research Index F8 condition (0.2 M ammonium sulfate, 0.1 M Hepes pH 7.5, and 25% polyethylene glycol 3350). Crystals of *At*SHMT4 were grown in 80% Hampton Research Index D9 condition (0.1 M Tris-HCl pH 8.5, and 25% polyethylene glycol 3350). Mature crystals were transferred to drops with the original screen conditions supplemented with 20% ethylene glycol, 50 mM L-serine, and 10 mM of MTX or PTX. After 24-h incubation, crystals were harvested and vitrified in liquid nitrogen. Data were collected at beamlines 22-BM and 22-ID beamlines at the Advanced Photon Source, Argonne, USA. The diffraction images were processed with *XDS*^56^. The statistics of data collection and processing are summarized in Table 3.

**Table 3:** Data collection and refinement statistics.

	*At*SHMT2-MTX	*At*SHMT4-MTX	*At*SHMT2-PTX
Data collection			
Beamline	APS 22-BM	APS 22-ID	APS 22-BM
Wavelength (Å)	1.0000	1.0000	1.0000
Space group	*P*2_1_2_1_2_1_	*P*2_1_2_1_2_1_	*P*2_1_2_1_2_1_
Unit cell parameters			
*a, b, c* (Å)	115.4, 130.7, 151.3	118.8, 120.9, 131.8	115.4, 130.8, 151.2
Resolution (Å)	80.0–1.63 (1.73–1.63)	80.0–2.12 (2.25–2.12)	80.0–1.54 (1.64–1.54)
Unique reflections	279297 (44247)	106878 (17038)	330027 (52224)
Multiplicity	8.2 (7.8)	4.2 (4.3)	8.1 (7.7)
Completeness (%)	99.8 (98.5)	98.8 (98.7)	99.7 (98.2)
*R*_meas_^a^(%)	9.3 (124.1)	10.8 (76.0)	8.8 (133.8)
<*I*/σ(*I)*>	18.3 (2.0)	11.6 (1.9)	18.88 (1.8)
Refinement			
*R*_free_ reflections	1396	1068	1320
No. of atoms (non-H)	17397	15779	17445
protein	15078	14583	15198
ligands	397	231	311
solvent	1922	965	1936
*R*_work_/*R*_free_ (%)	12.5/17.7	17.4/23.4	12.9/16.6
Average B-factor (Å^2^)			
protein	24.8	38.6	23.8
ligands	47.7	57.8	45.6
solvent	41.3	39.3	40.5
rmsd from ideal geometry			
bond lengths (Å)	0.005	0.006	0.005
bond angles (°)	0.81	0.90	0.79
Ramachandran statistics (%)			
favored	97.9	96.7	97.6
allowed	2.1	3.4	2.4
outliers	0.0	0.0	0.0
PDB ID	6smn	6smr	6smw

Values in parentheses correspond to the highest resolution shell.

^a^*R*_meas_ = redundancy independent R-factor.

### Determination and refinement of the crystal structures

Crystal structures were solved using molecular replacement in Phaser^57^ with *M. truncatula* SHMT3 as the search probe (PDB ID: 6cd0^7^). The models were built using *Phenix AutoBuild*^58^, and were placed inside the unit cells with the *ACHESYM* server^59^. *COOT*^60^ was used for manual fitting in the electron density maps between rounds of model refinement in *Phenix.refine*^61^. The refinement statistics are listed in Table 3. After a thorough quality validation with Molprobity^62^, the structures were deposited in the PDB^63^.

### Other software used

Molecular figures were created with UCSF *Chimera*^64^. Surface electrostatic potential was calculated using *PDB2PQR* and *APBS* servers^65,66^. Flat scheme of H-bonding network was created in *LigPlot+*^67^. Signal peptides were predicted with the *TargetP*^51^ webserver.

### Accession numbers

PDB IDs: *At*SHMT2-MTX complex, 6smn; *At*SHMT4, MTX complex, 6smr; *At*SHMT2-PTX complex, 6smw.

## Supplementary information

Supplementary Information
