# Supplementary material for: Structural basis of methotrexate and pemetrexed action on serine hydroxymethyltransferases revealed using plant models
Source: Sci Rep. 2019 Dec 23;9:19614. doi: 10.1038/s41598-019-56043-4 (PMC6928210; doi:10.1038/s41598-019-56043-4)
Supplement: Supplementary file 1 — Supplementary Information [file 41598_2019_56043_MOESM1_ESM.pdf]

Supplementary Information

Structural basis of methotrexate and pemetrexed action on serine hydroxymethyltransferases revealed using plant models

Milosz Ruszkowski[1,2]*, Bartosz Sekula[1], Agnieszka Ruszkowska[3,2], Roberto Contestabile[4], Isabel Nogues[5], Sebastiana Angelaccio[4], Andrzej Szczepaniak[6], and Zbigniew Dauter[1]

[1]Synchrotron Radiation Research Section of MCL, National Cancer Institute, Argonne, IL, USA

[2]Institute of Bioorganic Chemistry, Polish Academy of Sciences, Poznan, Poland

[3]Department of Chemistry and Biochemistry, University of Notre Dame, Notre Dame, IN, USA

[4]Department of Biochemical Sciences "A. Rossi Fanelli", Sapienza University of Rome, Laboratory affiliated to Istituto Pasteur Italia-Fondazione Cenci Bolognetti, P.le Aldo Moro 5, 00185 Roma, Italy

[5]Research Institute of Terrestrial Ecosystems, Italian National Research Council, Via Salaria Km. 29, 300–00015 Monterotondo Scalo, Roma, Italy

[6]Department of Hematology and Bone Marrow Transplantation, Poznan University of Medical Sciences, Poznan, Poland

*To whom the correspondence should be addressed:

Milosz Ruszkowski, Institute of Bioorganic Chemistry, Polish Academy of Sciences, Noskowskiego 12/14 Poznan, Poland; mruszkowski@ibch.poznan.pl

A

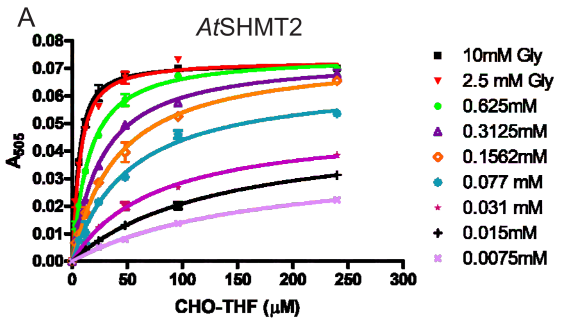

B

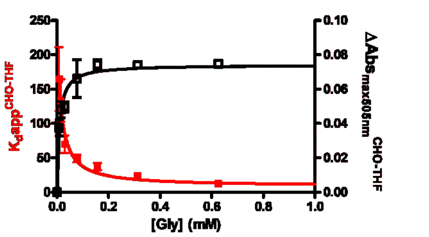

	Kd
Kd	253.6
aKd	8.262
K	0.01356
Std. Error	
Kd	38.39
aKd	4.717
K	0.004690

	Abs max
Absmax	0.07413
aKdGly	0.008770
Std. Error	
Absmax	0.001619
aKdGly	0.001257

Kd
Abs max

C

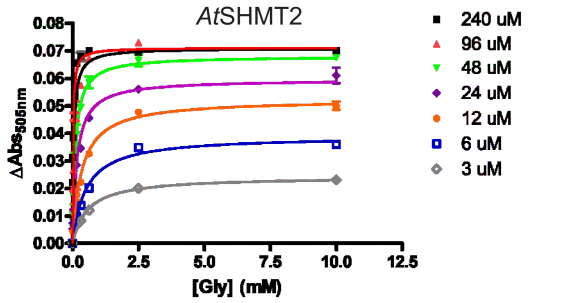

D

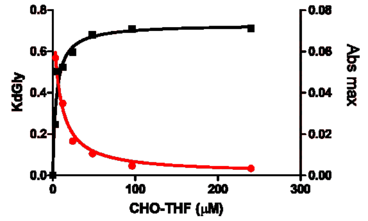

	Kd
Kd	0.8280
aKd	= 0.00877
K	7.523
Std. Error	
Kd	0.08428
K	1.728

	Abs max
Absmax	0.07308
aKdCHO	4.271
Std. Error	
Absmax	0.002637
aKdCHO	0.7723

E

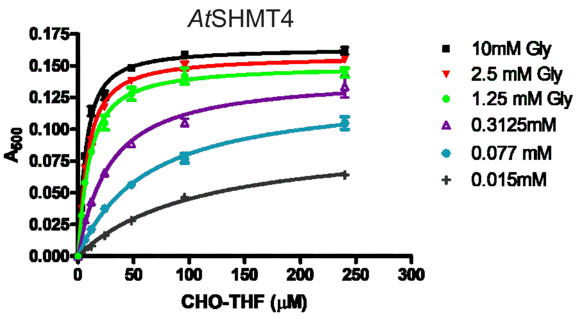

F

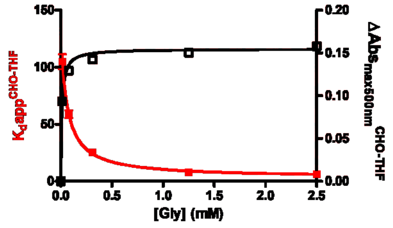

	Kd
Kd	129.3
aKd	3.080
K	0.06222
Std. Error	
Kd	2.714
aKd	0.8049
K	0.004319

	Abs max
Absmax	0.1547
aKdGly	0.01088
Std. Error	
Absmax	0.003341
aKdGly	0.001957

Kd
Abs max

G

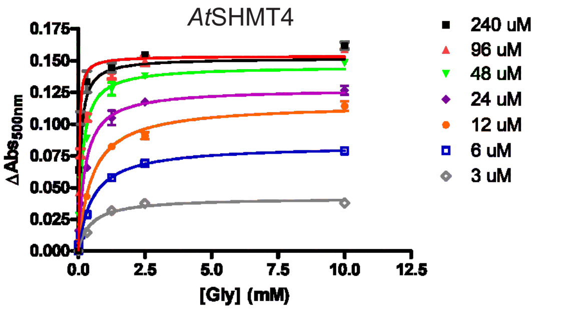

H

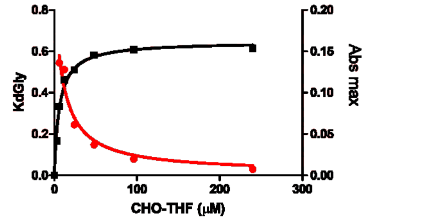

	Kd
Kd	0.9111
aKd	= 0.01088
K	10.30
Std. Error	
Kd	0.2103
K	4.762

	Abs max
Absmax	0.1617
aKdCHO	6.073
Std. Error	
Absmax	0.004400
aKdCHO	0.7429

Supplementary figure S1

Determination of glycine and 5-formyl-THF (CHO-THF) binding parameters. Panel A-B show *At*SHMT2 with variable 5-formyl-THF; C-D, *At*SHMT2 with variable glycine; E-F, *At*SHMT4 with variable 5-formyl-THF; G-H *At*SHMT4 with variable glycine. Assays were performed at 30 °C, in 20 mM KPi buffer at pH 8.0 for *At*SHM2 and pH 7.3 for *At*SHMT4. Absorbance was measured at 505 nm (*At*SHMT2) and 500 nm (*At*SHMT4) from the quinonoid intermediate formed upon addition of 5-formyl-THF to samples conteining protein (5 mM) and glycine. Samples with varied 5-formyl-THF (glycine at different fixed concentrations) and samples with varied glycine (5-formyl-THF at different fixed concentrations) were measured at equilibrium. The obtained saturation curves were fitted to obtained K_d and αK_d values as explained in Tramonti *et al.,* 2018.

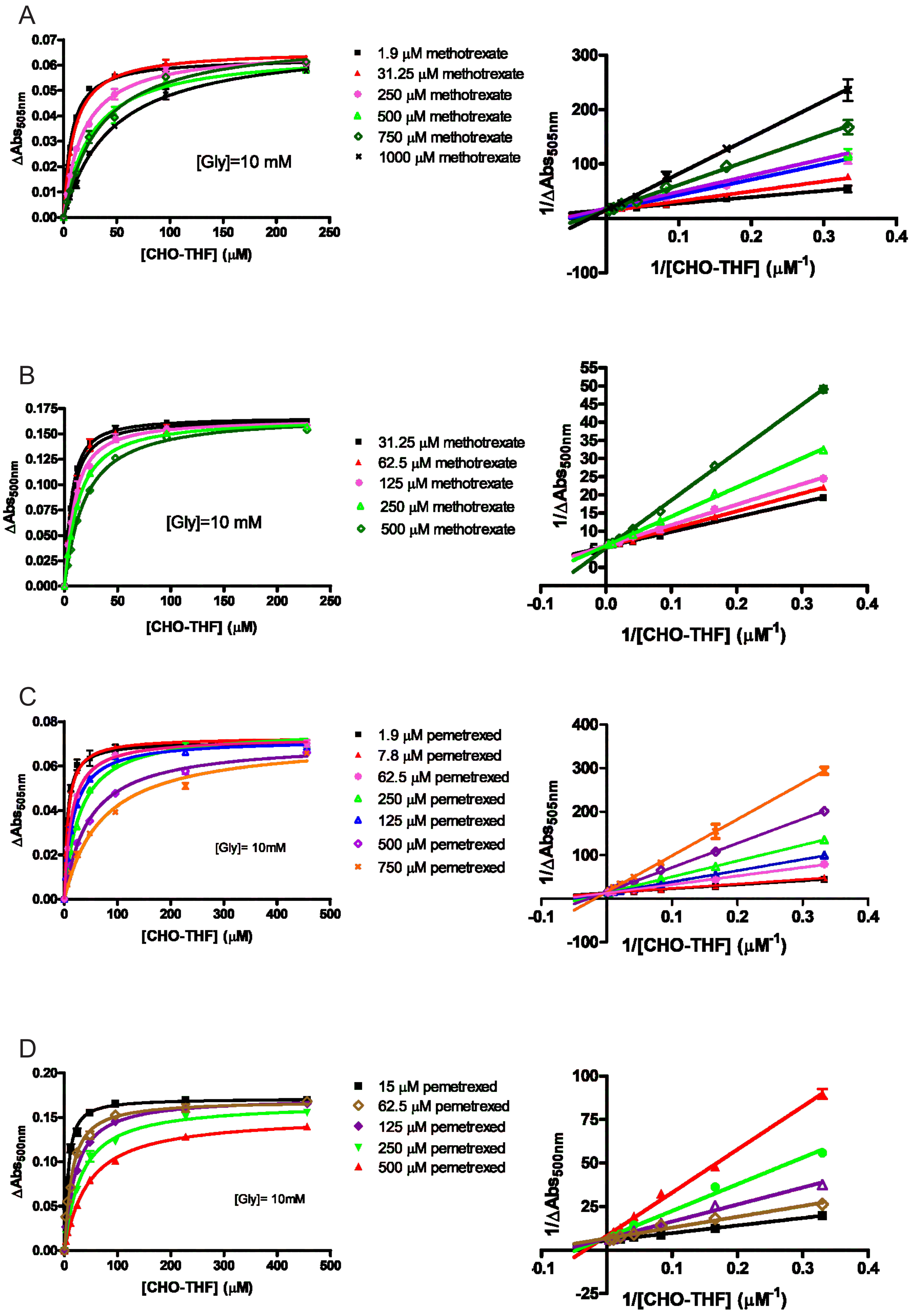

Supplementary figure S2

Methotrexate and pemetrexed inhibition experiments with *At*SHMT2 and *At*SHMT4. Panel A shows data for *At*SHMT2 inhibition by methotrexate; B, *At*SHMT4 by methotrexate; C, *At*SHMT2 by pemetrexed; D, *At*SHMT4 by pemetrexed. Absorbance at 505 nm (*At*SHMT2) or 500 nm (*At*SHMT4) from the quinonoid intermediate formed upon addition of 5-formyl-THF to samples containing enzyme (5 mM), glycine (10 mM), and the antifolate was measured at equilibrium. Right-side charts show double reciprocal plots of data shown in left-side panels fitted to a linear equation.
